# Zebrafish Larvae Carrying a Splice Variant Mutation in *cacna1d*: A New Model for Schizophrenia-Like Behaviours?

**DOI:** 10.1007/s12035-020-02160-5

**Published:** 2020-10-14

**Authors:** Nancy Saana Banono, Kinga Gawel, Linus De Witte, Camila V. Esguerra

**Affiliations:** 1 Chemical Neuroscience Group, Centre for Molecular Medicine Norway (NCMM), Faculty of Medicine, University of Oslo, Gaustadalléen 21, Forskningsparken, 0349 Oslo, Norway; 2 Department of Experimental and Clinical Pharmacology, Medical University of Lublin, Jaczewskiego Str. 8b, 20-090 Lublin, Poland; 3 Pharmaceutical and Biological Sciences, AP Hogeschool Antwerpen, Antwerp, Belgium; 4 School of Pharmacy, Faculty of Mathematics and Natural Sciences, University of Oslo, Sem Sælandsvei 24, 0371 Oslo, Norway

**Keywords:** Zebrafish, *CACNA1D*, Neurobehaviour, Neuropsychiatric disorders, Schizophrenia, Psychosis

## Abstract

Persons with certain single nucleotide polymorphisms (SNPs) in the *CACNA1D* gene (encoding voltage-gated calcium channel subunit alpha 1-D) have increased risk of developing neuropsychiatric disorders such as bipolar, schizophrenia and autism. The molecular consequences of SNPs on gene expression and protein function are not well understood. Thus, the use of animal models to determine genotype-phenotype correlations is critical to understanding disease pathogenesis. Here, we describe the behavioural changes in larval zebrafish carrying an essential splice site mutation (*sa17298*) in *cacna1da*. Heterozygous mutation resulted in 50% reduction of splice variants 201 and 202 (haploinsufficiency), while homozygosity increased transcript levels of variant 201 above wild type (WT; gain-of-function, GOF). Due to low homozygote viability, we focused primarily on performing the phenotypic analysis on heterozygotes. Indeed, *cacna1da*^*sa17298/WT*^ larvae displayed hyperlocomotion—a behaviour characterised in zebrafish as a surrogate phenotype for epilepsy, anxiety or psychosis-like behaviour. Follow-up tests ruled out anxiety or seizures, however, as neither thigmotaxis defects nor epileptiform-like discharges in larval brains were observed. We therefore focused on testing for potential “psychosis-like” behaviour by assaying *cacna1da*^*sa17298/WT*^ larval locomotor activity under constant light, during light-dark transition and in startle response to dark flashes. Furthermore, exposure of larvae to the antipsychotics, risperidone and haloperidol reversed *cacna1da*-induced hyperactivity to WT levels while valproate decreased but did not reverse hyperactivity. Together, these findings demonstrate that *cacna1da* haploinsufficiency induces behaviours in larval zebrafish analogous to those observed in rodent models of psychosis. Future studies on homozygous mutants will determine how *cacna1d* GOF alters behaviour in this context.

**Electronic supplementary material:**

The online version of this article (10.1007/s12035-020-02160-5) contains supplementary material, which is available to authorized users.

## Introduction

Schizophrenia (SCZ) is a highly heritable and polygenic neuropsychiatric disorder. The high prevalence of SCZ worldwide has made it a burgeoning public health concern. Genome-wide association studies (GWAS) have led to the initial identification of small nucleotide polymorphisms (SNPs) in about 108 risk loci linked to SCZ [1]. The majority of SNPs implicated in SCZ and other psychiatric disorders are located in noncoding (intergenic and intronic) regions of the genome [2]. To date, the functional significance of these noncoding variants remains unclear, thus obviating the need for establishment and validation of genetic animal models to elucidate underlying disease mechanisms associated with SCZ-risk genes. Nevertheless, SNPs in the intronic regions of genes are thought to result in changes in gene expression levels by altering splicing (*via *splice donor or acceptor sites) and transcription (*via *disruption of gene regulatory elements such as transcription factors, promoters, enhancers and/or suppressors) [3, 4]. For example, there is evidence that rs100637 (a SCZ-associated SNP, located in intron 3 of the *CACNA1C* gene) is associated with major depressive disorder (MDD), SCZ and attention deficit hyperactivity disorder, leads to changes in *CACNA1C* expression in both human carriers [5, 6] and induced human neurons [7]. In humans, broader phenotypes are present such as deficits in prepulse inhibition (PPI), latent inhibition and sleep disturbance in some SNP carriers, mimicking a broad spectrum of neuropsychiatric disorders [2, 8].

*CACNA1D* encodes the α1 subunit of the CaV_1.3_ voltage–gated L-type isoform. In the central nervous system (CNS) of humans, the CaV_1.3_ isoform is expressed in neuroendocrine cells, cerebral cortex, habenula, hippocampus, thalamus and basal ganglia, where it plays essential roles in consolidation of fear memory, drug-seeking behaviours and fine tuning of various elements of neuronal plasticity [9–12]. Mutations in *CACNA1D* have been implicated in neurodevelopmental and neuropsychiatric diseases [13–15]. For instance, persons with SNPs in *CACNA1D* have increased risk of developing bipolar disorder (BP), attention deficit hyperactivity disorder, SCZ, autism spectrum disorder (ASD) or MDD [1, 13, 16–21]. In one study, two single nucleotide variants in *CACNA1D* were found in separate cohorts of bipolar patients [22]. Although a study in the Han Chinese population did not find an association of *CACNA1D* with SCZ [23], other subsequent studies did identify such an association [8, 21]. Furthermore, whole-exome sequencing revealed variants in the *CACNA1D* coding region to be linked to ASD, depression, anxiety, fear and seizures [17, 18].

Homozygous CaV_1.3_ knockout mice are deaf and have impaired cardiac function [24], display antidepressant-like phenotypes [25] and have impaired consolidation of fear in the Pavlovian test [26]. In zebrafish (*Danio rerio*), *cacna1d* is duplicated into *cacna1da* and *cacna1db*, which encode CaV_1.3a_ and CaV_1.3b_ respectively [27]. The amino acid sequence of zebrafish Cacna1da and Cacna1db is 77% and 33% homologous to human CACNA1D respectively [28, 29]. Previous zebrafish studies have focused primarily on characterising the role of *cacna1d* in auditory and vestibular function [27, 30–32]. Homozygous *cacna1da* nonsense mutants display a classical auditory-vestibular phenotype reminiscent of “circler mutants” (i.e. circular swimming due to impaired balance) [27, 33, 34].

Interestingly, the essential splice site mutation described in this study was found to mimic both gene haploinsufficiency in the heterozygous state and gain-of-function (GOF) in the homozygous state. As homozygous mutant survival was low (i.e. very small sample size), we focused the majority of our analysis on heterozygotes, thus investigating the effects of *cacna1da* haploinsufficiency on larval zebrafish behaviour and electroencephalography (EEG) patterns. We profiled for anxiety-, seizure- or psychosis-like behaviours, by performing the following assays: PPI response, locomotor activity (constant light), light-dark transition test, thigmotaxis and startle response to dark flashes. We also tested the neuromodulatory activity of risperidone (RISP), an atypical antipsychotic drug [35]; haloperidol (HALO), a dopamine antagonist used as a typical antipsychotic drug [36]; and valproic acid (VPA), an anti-seizure drug and mood stabiliser [37, 38], to determine whether *cacna1d* mutant larvae would respond in a similar manner as SCZ, BP and/or epilepsy patients/animal models. Finally, EEG recordings were performed to establish whether the hyperlocomotion observed at earlier developmental time points resulted from epileptiform-like brain activity.

## Materials and Methods

### Ethical Considerations

Approval by the Norwegian Food Safety Authority *via *its experimental animal administration’s supervisory and application system (FOTS-ID 15469 and 23935) was obtained prior to animal experimentation. Also, compliance with the National Institute of Health Guidelines for the Care and Use of Laboratory Animals and the European Community Council Directive of November 2010 for Care and Use of Laboratory Animals (Directive 2010/63/EU), as well as the ARRIVE guidelines, were adhered to during all experiments.

### Zebrafish Strains and Husbandry

The *sa17298* mutant line was generated by N-ethyl-N-nitrosourea (ENU) mutagenesis within the Zebrafish Mutation Project (Sanger, UK). The line carries a point mutation in *cacna1da*, which spans an essential splice site [for details see: [39]]. Fertilised *sa17298* embryos were obtained from the Zebrafish International Resource Center (Eugene, Oregon, USA) and raised to adulthood, genotyped and the heterozygous animals outcrossed to AB wild-type (WT) zebrafish for three generations. Animals were raised under controlled conditions described by [40], in a 14-/10-h light/dark cycle at 28.5 °C.

Fertilised eggs from natural spawning of adult fish lines were collected, transferred to petri dishes (density *N* = 50–70), filled with embryo medium (17 mM NaCl, 2 mM KCl, 1.8 mM Ca(NO3), 2,0.12 mM MgSO4, 1.5 mM HEPES buffer pH 7.1–7.3 and 0.6 μM methylene blue). These petri dishes were stored in an incubator with a 14/-10-h light/dark cycle at 28.5 °C. The medium was refreshed daily until larvae reached 7-day post-fertilisation (dpf).

### Genotyping

Adult fin clip tissue or whole larvae were snap frozen in liquid nitrogen. DNA from fin clip tissue or larval sample was extracted using PCR extraction buffer (10 mM Tris (pH 8.0), 2 mM EDTA, 0.2% Triton X-100) and proteinase K (200 μg/ml). DNA was amplified using DreamTaq DNA polymerase (EP0702, Thermofischer) according to the manufacturer’s instructions in a 20 μL final volume. The forward primer: 5′ *TGTGCTGGTGTTGTGTGTG 3′* and reverse primer: 5′ *TCAAGCCAGGAAGTACTGAAG 3′* were used with the following cycling conditions: Step 1: Initial denaturation 95 °C, 1 min; Step 2: Denaturation 95 °C, 30 s; Step 3: Annealing temperature 59 °C, 30 s; Step 4: Extension 72 °C, 1 min; Step 5: Repeat steps 2–4, 34 ×; Step 6: Final extension 72 °C, 1 min. This resulted in a 196 base pair (bp) amplicon. The PCR product was digested using the restriction enzyme, BstEII (R0162M, New England BioLabs). The bands were visualised using 2% agarose gel electrophoresis and Sybr Safe (S33102, Thermofischer) as the DNA intercalating agent. Observers conducting behavioural tests were blind to the genotype of the larvae. Hence, larvae were genotyped after each experiment.

### Morphological Assessment

Larvae were photographed using a Leica M205 FA stereomicroscope and assembled using Adobe Photoshop 2020. All pictures were taken at 5 dpf at the same resolution for comparison.

### Drugs

The following drugs were used: 5 μM RISP, 50 μM HALO and 100 μM VPA. All drugs were dissolved in DMSO at a final concentration of 0.5% v/v DMSO for the HALO and VPA groups but 0.1% v/v DMSO for the RISP-treated group. The appropriate vehicle controls were prepared by dissolving DMSO in zebrafish E3 medium. DMSO and HALO were purchased from Sigma-Aldrich, RISP from TOCRIS and VPA from Sanofi Aventis. The drug concentrations used were selected based on previously published studies [35, 36, 41].

### Behavioural Tests and Material

Larvae were transferred to the behavioural analysis room at least 1 h prior to experimentation to allow animals to acclimate. Single larvae were gently transferred from the petri dishes to individual wells of a 24-well plate (diameter 16.2 mm) except when stated otherwise. Automated video-tracking of larval behaviour was carried out using the ZebraBox hardware and ZebraLab software (Viewpoint, Lyon, France). Light-dark test and thigmotaxis measurement were carried out using the same larvae by simply defining the inner and outer regions of each well of the tracking plate (the inner zone was of diameter 8 mm while the distance from the inner zone relative to the outer zone was 4 mm). A different set of larvae were used for the startle response test. Except for the startle response to dark flashes test, all other experiments were replicated two or three times and the results pooled together.

### Locomotor Activity and Thigmotaxis

Larvae were allowed to acclimate to the test chamber (ZebraBox) for 15 min in the dark, followed by 10 min of tracking in either 100% light or dark (0% light). For the light-dark transition test, after the acclimation, 10 min each of tracking in the following conditions: (1) 100% light followed by (2) dark in succession. The locomotor activity of larvae was measured as the total distance travelled in millimetres (mm) over a 10-min period.

For thigmotaxis, the following parameters were measured: (1) distance spent in inner zone, (2) distance spent in outer zone and (3) distance in entire arena. Thigmotaxis was calculated as percentages using the following formula [42]:

$$ \mathrm{Thigmotaxis}\ \left(\%\mathrm{distance}\ \mathrm{in}\ \mathrm{outer}\ \mathrm{zone}\right)=\frac{\left(\mathrm{Distance}\ \mathrm{outer}\ \mathrm{zone}\right)}{\left(\mathrm{Distance}\ \mathrm{outer}+\mathrm{inner}\ \mathrm{zone}\right)}\times 100 $$

### Startle Response to Dark Flashes

To trigger a startle response in larvae, 150-ms-long dark flashes were used, and this was repeated severally to assess the ability of the larvae to habituate. The startle response test was made up of three steps: (1) 15-min acclimatisation period in 100% illuminated test chamber, (2) 10-min baseline locomotor tracking in the fully illuminated test chamber, (3) followed by the presentation of 30 dark flashes repeated every 3 s [43].

### Locomotor Activity of Drug-Treated Larvae

Larvae were pre-exposed 30 min to RISP, HALO or VPA and their respective vehicle control groups, transferred to the test chamber and tracking started immediately for 4 h at a 30 -min integration period. Only tracks after 2 h were used for analysis. For the 24-h RISP-treated group, larvae were pre-exposed to treatment 22 h then transferred to the test chamber and tracked immediately for 4 h with the tracks after 2 h used for analysis. Larvae were tracked in 100% light condition and the locomotor activity of larvae were measured as the total distance travelled in millimetres over a 30-min period. Vehicle control and drug-treated larvae were from the same clutch.

### Acoustic Startle Response (ASR) and Prepulse Inhibition (PPI)

Acoustic stimuli were delivered using the ZebraBox Revo and the behavioural response analysed with EthoVision software as previously described [44] using larvae aged 6 dpf. All experiments were performed by placing larvae individually in each well of a custom-made plexiglass plate of 33 wells in a 96 format. Acclimation was for 5 min prior to the onset of experiment in a 100-Lx illuminated chamber. The startle stimulus was 100 ms at 660 Hz while the prepulse stimulus was 5 ms at 440 Hz. A 100-ms inter-stimulus interval (ISI) was used for PPI experiments.

### Zebrafish EEG Recordings

The EEG recordings were performed as previously described by Afrikanova et al. [45]. Epileptiform-like discharges were detected by inserting a glass electrode filled with artificial cerebrospinal fluid (124 mM NaCl, 2 mM KCl, 2 mM MgSO_4_, 2 Mm CaCl_2_, 1.25 mM KH_2_PO_4_, 26 mM NaHCO_3_, 10 mM glucose) into the optic tectum of individual 6-dpf zebrafish larvae for 20 min (MultiClamp 700B amplifier, Digidata 1550 digitiser, Axon instruments, USA). The larvae were restrained with the aid of a thin layer of 2% low melting point agarose. The Clampfit version 10.6.2 software (Molecular Devices Corporation, USA) was used for processing the EEG recordings. The data were analysed manually by a trained observer, blind to the genotype of the larvae.

### RNA Isolation and cDNA Synthesis

Total RNA was isolated from a pool of 6-dpf larvae (*N* = 25 larvae, 3 replicates/group) using the Invitrogen PureLink RNA Mini Kit (12183018A, Thermofischer). A Nanodrop 1000 spectrophotometer and agarose bleach gel described by [46] was used to assess the quality/integrity of the RNA. cDNA was synthesised from 1 μg RNA with oligo dT primers using the Invitrogen SuperScript IV Reverse Transcriptase (18090050, Thermofischer) according to the manufacturer’s instructions.

### Reverse Transcriptase and Quantitative Polymerase Chain Reaction

rt-PCR was performed for 40 cycles using Taq-polymerase (EP0406, Thermofischer) in a 20 μl final reaction using 1 μl of cDNA template. PCR products were visualised on 2% agarose gels. The qPCR reactions were performed with Power SYBR Green Master Mix (4368702, Thermofischer) on the CFX384 Touch Real-Time PCR Detection System (1855485, Bio Rad). All primers were synthesised by Sigma with melting temperature > 60 °C. Samples were run in triplicates in a 20 μl final volume containing 10 μl 2 × sybr green, 1 μl 10 μM forward + reverse primer and 9 μl 1:180 cDNA dilution. The following cycling parameters were used: 95 °C for 10 min, and then 40 cycles of 95 °C for 15 s, 60 °C for 1 min. To check for the presence of non-specific products and/or primer dimers, a dissociation step was performed at the end of each amplification phase from 65 to 95 °C, at 0.5 °C increment for 5 s. The comparative ΔΔCT method was calculated using the Bio Rad CFX Manager 3.1. The endogenous genes *glyceraldehyde-3-phosphate dehydrogenase* (*gapdh*) and *ribosoamal protein S18 (rps18)* were used for data normalisation. See Suppl. Table 1 for the list of rt-/q-PCR primer sequences.

### Statistical Analysis

Statistical analysis and figures generation were performed with the aid of GraphPad Prism 8.4.1 (San Diego, CA, USA). For behavioural experiments, a two-way analysis of variance (ANOVA) (factors: phase/treatment and genotype), followed by Tukey’s *post hoc *test, was used. For EEG experiment, Student’s *t* test was applied. Unpaired Student’s *t* test or its equivalent non-parametric test, Mann Whitney *U*, was performed where necessary. Statistical significance was established at *p* < 0.05. In figures, dots represent individual measurements.

## Results

### Genotyping and Molecular Consequences of the *cacna1da* Splice Site Mutation

**Fig. 1:**
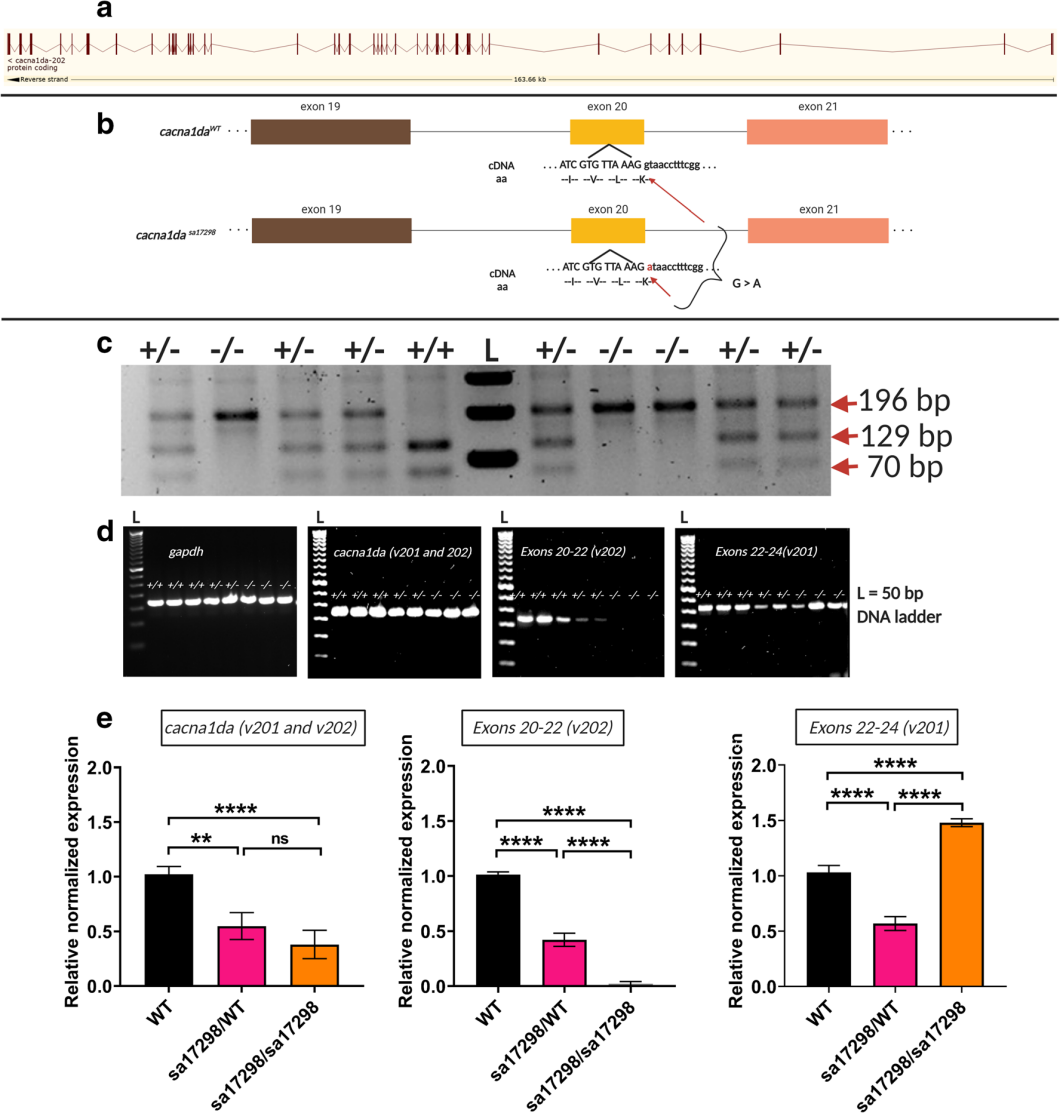
Zebrafish *cacna1da* mutant allele (*sa17298*) and its molecular consequence. **a** Schematic representation of the zebrafish *cacna1da* transcript. Red bars represent exons and lines represent introns. Image retrieved from ensembl.org. **b** Schematic representation of zebrafish *cacna1da* and *sa17298* mutation. Nucleotides in upper case are within the exon while those in lower case are within the intron. **c** Sample gel electrophoresis of BstEII restriction digest and resulting PCR products to determine genotype of fish/larvae. BstEII does not cut homozygous *sa17298*, but cuts WT into two bands (70 and 119 bp) and heterozygous *sa17298* into three bands (70, 119 and 196 bp). Note the multiple bands in the BstEII digested *versus *the undigested corresponding samples. L: 1 kilobase DNA ladder, +/+: *cacna1da*^*WT/WT*^, +/−: *cacna1da*^*sa17298/WT*^, −/−: *cacna1da*^*sa17298/sa17298*^*.* **d** *cacna1da* mRNA levels as measured by agarose gel electrophoresis (rt-PCR). **e** *cacna1da* mRNA levels as measured by qPCR. *cacna1da* mRNA expression is normalised against *gapdh* and *rps18*

As mentioned earlier, the amino acid sequence of zebrafish Cacna1da and Cacna1db are 77% and 33% homologous to human CACNA1D protein respectively. *cacna1da* has two transcript variants while *cacna1db* has a single transcript [28, 29]. The cacna1da transcript variant 202 spans 47 exons (Fig. 1a), whereas variant 201 spans 48 exons (not shown). The *sa17298* allele contains a single point mutation (G > A) at the first residue of intron 20–21 of the cacna1da*-202* transcript variant (Fig. 1b). Importantly, it does not alter the sequence of variant 201. To identify mutants, PCR of larval and/or adult fin clip tissue was digested using BstEII restriction enzyme. The BstEII restriction site is absent in the mutant allele. Hence, 2% agarose gel electrophoresis of the digested product resulted in one band for homozygous *sa17298* (~ 196 bp), two bands for WT *sa17298* (~ 70 and 129 bp) and three bands for heterozygous *sa17298* (~ 70, 129 and 196 bp) (Fig. 1c).

To assess whether the splice site mutation resulted in exon skipping, rt-PCR, followed by agarose gel electrophoresis, was performed using primers spanning exons 20–22 of cacna1da transcript variant 202. No difference in product size was observed between WT and homozygous mutants, thus suggesting that the mutation did not result in exon skipping. Notably, however, a difference in signal intensity of the rt-PCR amplicons was observed (Fig. 1d). Given the difference in signal intensity obtained from WT and homozygotes, we next tested whether the mutation altered mRNA levels. We performed qPCR analysis using three *cacna1da* primer sets. The first primer set [cacna1da_qPCR] amplified a region with high complementarity between the 201 and 202 transcript variants, the second primer set [E20-22_v202] targeted only the 202 transcript while the third primer set [E22-24_v201] targeted only the 201 transcript (Suppl. Fig. 1). qPCR analysis revealed an overall reduction of *cacna1da* mRNA levels (ca. 50% and 40%) in heterozygous and homozygous mutants respectively. Primer-specific amplification of variant 202 showed a 50% and 90% decrease in transcript 202 levels in heterozygous and homozygous mutants respectively. Interestingly, when primers specifically targeting transcript variant 201 were used, homozygous mutants showed a 50% increase, while heterozygous mutants showed a 50% reduction in levels of variant 201 (Fig. 1e).

### Morphological Analysis of *sa17298* Mutants

**Fig. 2:**
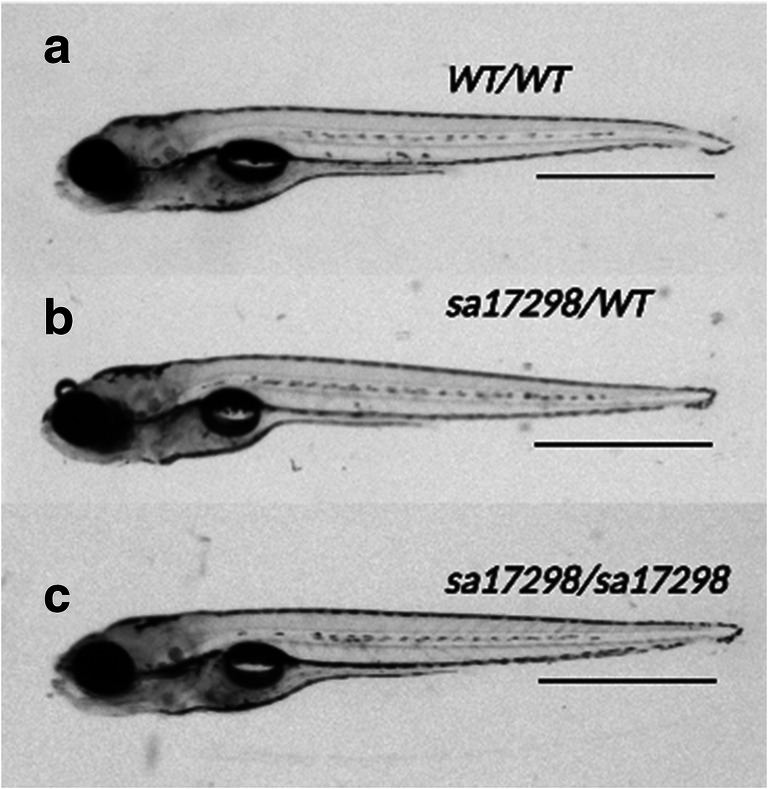
Morphology of **a** WT *cacna1da*^*WT/WT*^ **b** heterozygous *cacna1da*^*sa17298/WT*^ and **c** homozygous *cacna1da*^*sa17298/sa17298*^. Both heterozygous and homozygous mutants are morphologically indistinguishable from WT siblings. Scale bar (a–**c**): 1 mm

When AB-outcrossed mutants were bred, the resulting heterozygous and homozygous mutants were morphologically indistinguishable from their WT siblings (Fig. 2). All experiments were carried out using the AB outcrossed fish.^1^ Based on genotyping results, less than 5% of homozygous *sa17298* larvae survived until adulthood. No impairment in the touch-evoked response of heterozygous and homozygous *sa17298* larvae was observed when compared to WT (data not shown).

### Acoustic Startle Response and Prepulse Inhibition

**Fig. 3:**
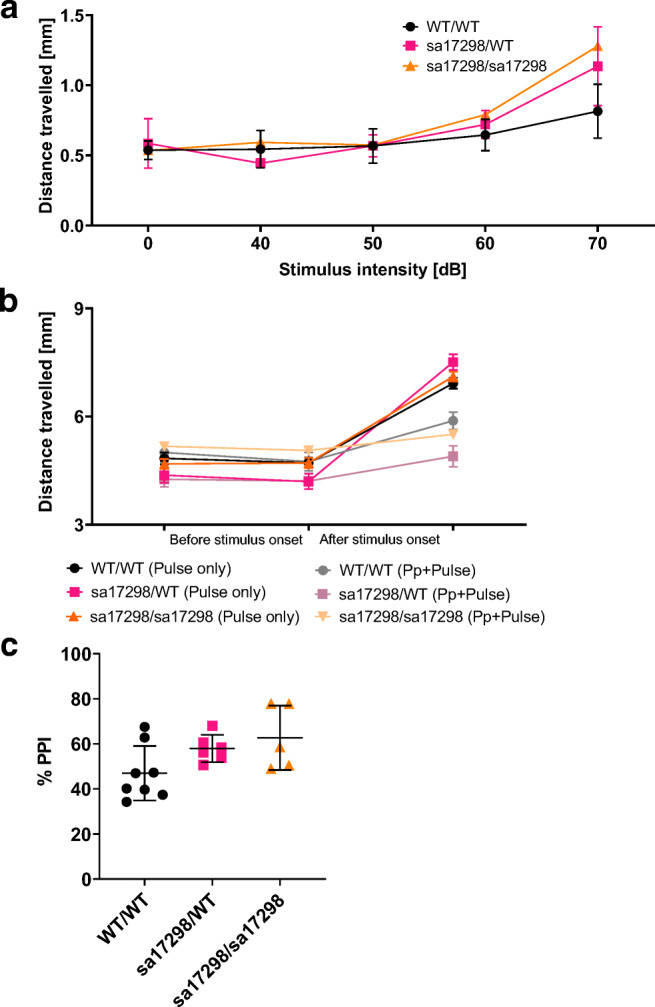
Acoustic startle response and PPI. **a** Graph of distance travelled when presented with different auditory stimuli. Data analysed using two-way ANOVA and presented as mean ± SEM. **b** Preceding a startle with a prepulse stimulus decreases the startle response of larvae. Data shown as mean ± SEM. **c** % PPI of larvae. One-way ANOVA revealed no overall significant difference among the groups. Tukey’s multiple comparison *post hoc *test however showed a strong tendency of homozygous mutant larvae towards significance (i.e. enhanced PPI). *cacna1da*^*WT/WT*^ (*N* = 8), *cacna1da*^*sa17298/WT*^ (*N* = 6) and *cacna1da*^*sa17298/sa17298*^ (*N* = 5), *p* > 0.05. Error bars represent mean ± SD. ASR, acoustic startle response; Pp, prepulse; PPI, prepulse inhibition. *cacna1da*^*WT/WT*^ vs *cacna1da*^*sa17298/WT*^*: p* = 0.2002, *cacna1da*^*WT/WT*^ vs *cacna1da*^*sa17298/sa17298*^*: p* = 0.0646, *cacna1da*^*sa17298/WT*^ vs *cacna1da*^*sa17298/sa17298*^*: p* = 0.7702

Four different auditory stimuli (40, 50, 60, 70 dB) at 660 Hz were tested to determine the suitable startle stimuli, and it was determined that 70 dB evoked the strongest startle response at *p* < 0.05 (Fig. 3a). When a 50-dB prepulse preceded the 70-dB stimulus, larval startle response was decreased (Fig. 3b). One-way ANOVA revealed no statistically significant difference in the PPI (%) between WT and either mutant genotype (i.e. heterozygotes and homozygotes) (Fig. 3c) [*F* (2, 16) = 1.403, *p* = 0.2745]. Notably, however, there was a tendency of homozygotes towards statistical significance (i.e. increased PPI).

### Locomotor Assessment of *sa17298* Larvae

**Fig. 4:**
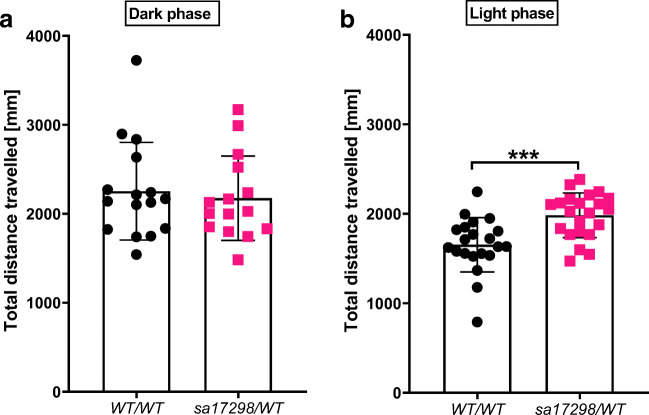
Locomotor activity of WT and heterozygous *sa17298* zebrafish larvae at 6 dpf, tracked independently under different illumination conditions. **a** Tracking in the dark reveals no difference in locomotor activity between *cacna1da*^*WT/WT*^ (*N* = 16) and *cacna1da*^*sa17298/WT*^ (*N* = 15). **b** Tracking in 100% light reveals a statistically significant difference in locomotor activity between *cacna1da*^*WT/WT*^ (*N* = 21) and *cacna1da*^*sa17298/WT*^ (*N* = 22). ***p < 0.001. Data analysed using Mann Whitney *U* test and represented as mean ± SD

To determine if the *sa17298* mutation modulates locomotor behaviour, one batch of larvae were tracked in dark conditions (0% light) while another group of larvae were tracked in 100% light. Mann Whitney *U* test showed no statistically significant difference between the genotypes [*cacna1da*^*WT/WT*^ (Mdn = 2155, *N* = 16) and *cacna1da*^*sa17298/WT*^ (Mdn = 2027, *N* = 15); *U* = 109, *p* = 0.6823] when tracked in the dark (Fig. 4a). On the other hand, when larvae where tracked in 100% light conditions, Mann Whitney *U* test indicated that the total distance travelled by *cacna1da*^*WT/WT*^ (Mdn = 1632, *N* = 21) was significantly lower than *cacna1da*^*sa17298/WT*^ (Mdn = 2039, *N* = 22); *U* = 92, *p* = 0.0005] (Fig. 4b).

### Larval Behaviour in Response to Abrupt Change in Illumination

**Fig. 5:**
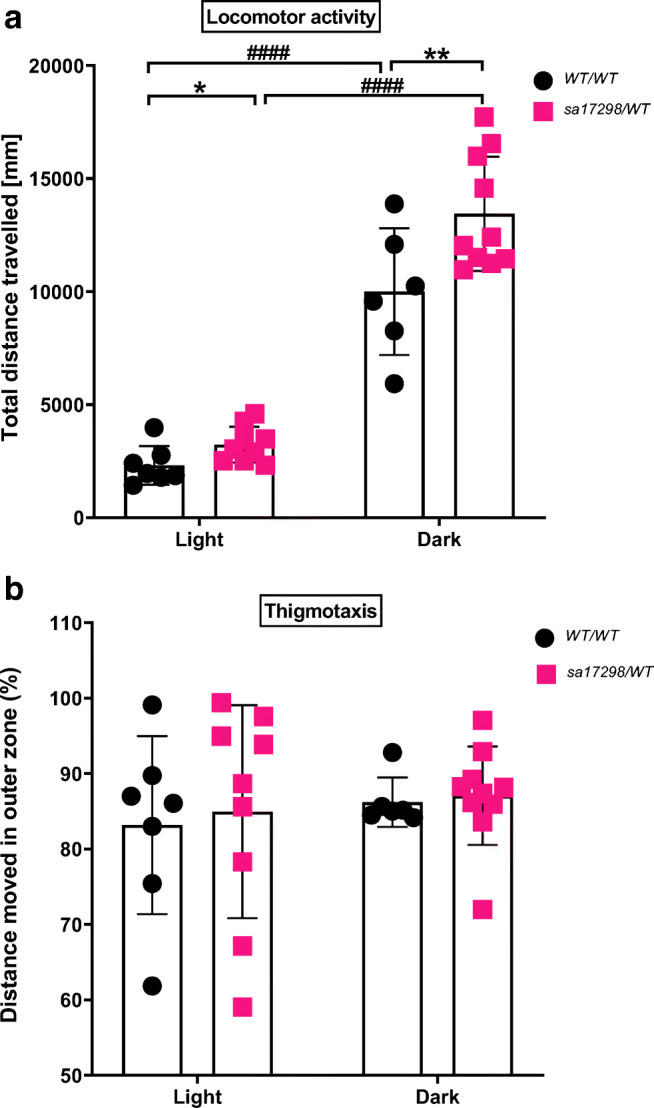
Behaviour of WT and heterozygous *sa17298* zebrafish larvae at 6 dpf in the light-dark transition test. Each dot represents an individual larval measurement. Data analysed using non-RM two-way ANOVA followed by Tukey’s *post hoc *test. Data represented as mean ± SD. **a** Light-dark transition elicits an increase in locomotor activity in both genotypes with locomotor difference between *cacna1da*^*WT/WT*^ and *cacna1da*^*sa17298/WT*^ reaching statistical significance in both illumination states. * (*p* < 0.05), ** (*p* < 0.01): [*cacna1da*^*WT/WT*^ vs *cacna1da*^*sa17298/WT*^] in the dark, #### (*p* < 0.0001): [*cacna1da*^*WT/WT*^ vs *cacna1da*^*WT/WT*^] and [*cacna1da*^*sa17298/WT*^ vs *cacna1da*^*sa17298/WT*^]. **b** Zone preference of WT and heterozygous *sa17298* larvae (thigmotaxis) represented as % total distance moved in the outer zone in the light-dark transition test

The locomotor behaviour of larval zebrafish differs depending on light-dark transitions [47]. Based on the observations made when two groups of larvae were independently tracked in either light or dark conditions, we hypothesised that changes in illumination in the tracking chamber would alter locomotor activity of *sa17298* larvae. We analysed spontaneous swimming after a 15-min acclimation period. Swimming in each illumination state (light-dark) was recorded for 10 min (Fig. 5).

In Fig. 5 a, larvae demonstrated an increased locomotor activity when transitioned from light to dark shown by a non-RM two-way ANOVA analysis [genotype *F* (1, 28) = 9.645, *p* < 0.01; illumination *F* (1, 28) = 162.3, *p* < 0.0001; interaction *F* (1, 28) = 3.241, *p* = 0.0826]. Tukey’s *post hoc *test revealed that both *cacna1da*^*WT/WT*^ (*p* < 0.0001) and *cacna1da*^*sa17298/WT*^ (*p* < 0.0001) travelled higher distances in the dark than in the light. Additionally, the *post hoc *test indicated statistical significance between *cacna1da*^*WT/WT*^ and *cacna1da*^*sa17298/WT*^ when they transitioned to the dark (*p* < 0.01), whereas Student’s *t* test showed statistical significant difference between *cacna1da*^*WT/WT*^ and *cacna1da*^*sa17298/WT*^ in the light phase (*p* < 0.05).

A previous study reported that changes in illumination altered the zone preference of larvae in the thigmotaxis test [42]. Therefore, we sought to determine if changes in illumination modulated the zone preference of *cacna1da*^*WT/WT*^ and *cacna1da*^*sa17298/WT*^ larvae. Larvae were assessed for the distance travelled (%) in the outer zones of a 24-well plate arena within 10 min.

As seen in Fig. 5b, there was no significant difference in the zone preference of *cacna1da*^*WT/WT*^ and *cacna1da*^*sa17298/WT*^ larvae using non-RM two-way ANOVA analysis in the light-dark transition [genotype *F* (1, 28) = 0.1310, *p* = 0.7201; illumination *F* (1, 28) = 0.4996, *p* = 0.4855; interaction *F* (1, 28) = 0.01618, *p* = 0.8997].

### Assessment of Startle Response to Repetitive Dark Flashes

Zebrafish larvae respond to startle stimuli such as unexpected light or sound stimulus and quantifying changes in distance travelled have been established as a suitable readout of startle response [43, 48]. Following the “startle response to a dark flash” protocol of Norton, larvae were presented with 30 dark flashes repeated every 3 s [43].

**Fig. 6:**
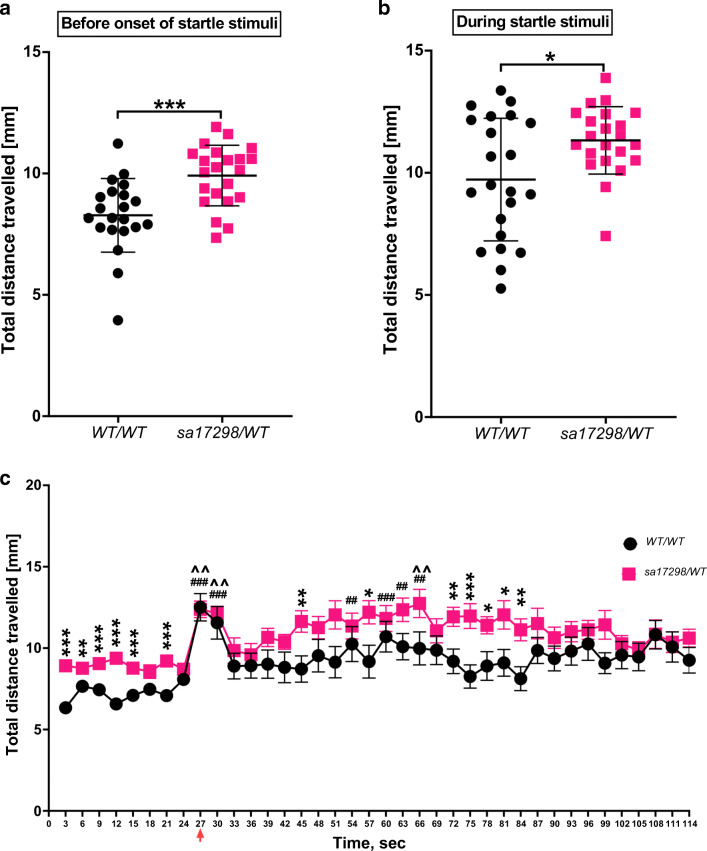
Startle response to dark flashes. **a** Baseline locomotor activity 21 s prior to onset of startle stimuli, ***p < 0.001. Data represented as mean ± SD. **b** Mean distance moved in response to startle stimuli. Unpaired Student’s *t* test showed *cacna1da*^*WT/WT*^ moved less than *cacna1da*^*sa17298/WT*^ during the 90-s startle stimulation, *p < 0.05. Data represented as mean ± SD. **c** Distance moved in response to startle stimuli represented as mean ± SEM. Paired Student’s *t* test analysis between *cacna1da*^*WT/WT*^ and *cacna1da*^*sa17298/WT*^ with statistical significance represented as *p < 0.05, ^**#**^*p* < 0.01, **^***p* < 0.001. Red arrow: onset of startle stimulus (dark flashes)

The locomotor activity of the last 21 s of the 10-min basal locomotor activity was measured preceding the dark flashes that were every 3 s thirty times (Fig. 6a). In general, *cacna1da*^*WT/WT*^ and *cacna1da*^*sa17298/WT*^ larvae neither swum the same distance during 21-s basal locomotor activity [*t* (41) = 3.879, *p* = 0.0004, Fig. 6a] nor the 90-s startle experiment [*t* (41) = 2.627, *p* = 0.0120, Fig. 6b].

The distance moved in response to the first and second dark flash was greater than the distance moved in response to subsequent stimuli. Whereas WT larvae showed signs of habituation after the 2nd stimulus, habituation was only visible after the 19th stimulus in heterozygous mutants. Multiple *t* test analysis showed that at several time points, *cacna1da*^*sa17298/WT*^ larvae were significantly more active than *cacna1da*^*WT/WT*^ at *p* < 0.05. Overall, *cacna1da*^*WT/WT*^ displayed a robust startle response relative to baseline than *cacna1da*^*sa17298/WT*^ [i.e. when we compared the last 21 s prior to stimulus onset with startle stimuli response] (Fig. 6c).

### Effects of Neuroactive Drugs on the Locomotor Activity of WT and Heterozygous *sa17298* Zebrafish Larvae

**Fig. 7:**
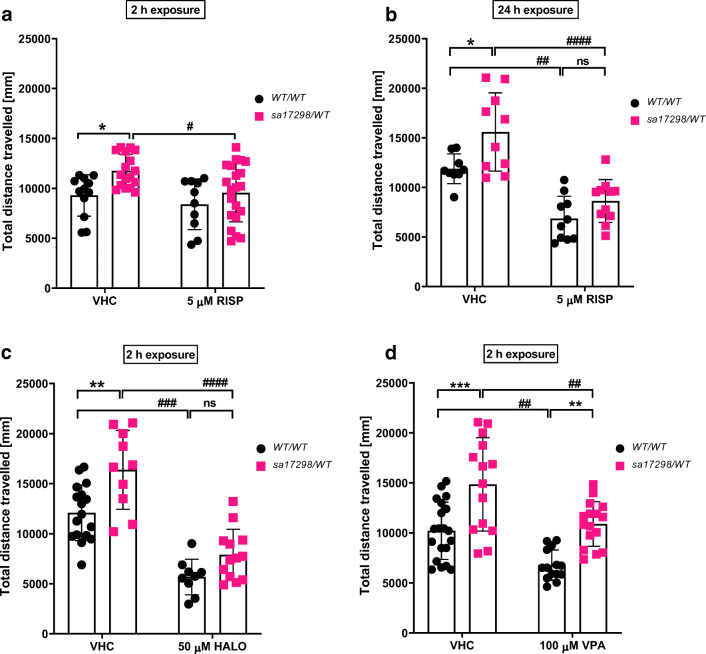
Effects of neuroactive drugs on the locomotor activity of 6-dpf WT and heterozygous *sa17298* larvae. Larvae were exposed to different neuroactive drugs. Each dot represents individual larval measurement. Data analysed using non-RM two-way ANOVA followed by Tukey’s *post hoc *test. Data represented as mean ± SD. **a** 2-h RISP, **b** 24-h RISP **c** 2-h HALO and **d** 2-h VPA. HALO, haloperidol; RISP, risperidone; VPA, valproic acid. *p < 0.05, **p < 0.01, ***p < 0.001 [*cacna1da*^*WT/WT*^ vs *cacna1da*^*sa17298/WT*^] in respective groups. ^**#**^*p* < 0.05, ^**##**^*p* < 0.01, ^**###**^*p* < 0.001, ^####^*p* < 0.0001 [*cacna1da*^*WT/WT*^ vs *cacna1da*^*WT/WT*^] and [*cacna1da*^*sa17298/WT*^ vs *cacna1da*^*sa17298/WT*^]

In zebrafish, hyperlocomotion is often characterised as a phenotype of epilepsy- [45, 49–52], anxiety- [51, 53] or psychosis-like behaviour [51, 54, 55]. To understand the possible cause of the hyperlocomotor behaviour, we observed in heterozygous *sa17298/WT* larvae, three neuroactive drugs i.e. RISP, HALO and VPA were evaluated for their ability to modulate the larval hyperactivity after 30 min of tracking. Non-RM two-way ANOVA revealed a significant effect of both genotype [*F* (1,56) = 8.010, *p* = 0.0064] and treatment [*F* (1,56) = 5.799, *p* = 0.0193] on locomotor activity when larvae were exposed to 5 μM RISP for 2 h. No significant interaction [*F* (1,56) = 1.066, *p* = 0.3064] was observed. However, a Tukey’s *post hoc *test showed that RISP was only effective in decreasing the locomotor activity of *sa17298/WT* (*p* < 0.05) but not WT/WT (*p* > 0.05) when compared with their untreated control (Fig. 7a).

To ascertain whether a prolonged exposure of zebrafish larvae to RISP would enhance RISP’s effectiveness in affecting locomotor activity, a set of larvae were incubated in RISP for 24 h and the locomotor activity measured subsequently. As seen in Fig. 7b, non-RM two-way ANOVA showed an overall statistically significant effect of RISP on larval locomotor activity after 24-h pre-exposure [genotype [*F* (1,36) = 10.77, *p* = 0.0023]; treatment [*F* (1,36) = 51.41, *p* < 0.0001]; interaction [*F* (1,36) = 1.351, *p* = 0.2527]]. Tukey’s post *hoc test *revealed a statistically significant decrease in locomotor activity in larvae treated with 5 μM RISP when compared with their untreated control at least *p* < 0.01 such that baseline locomotor activity became comparable between 5 μM [WT/WT vs *sa17298/WT*] at *p* = 0.4262.

Furthermore, two-way non-RM ANOVA of larvae exposed to 50 μM HALO for 2 h (Fig. 7c) yielded a statistically significant effect of genotype [*F* (1,45) = 15.03, *p* = 0.0003] and treatment [*F* (1,45) = 78.55, *p* < 0.0001] on locomotor activity with no significant interaction [*F* (1,45) = 1.518, *p* = 0.2243]. Subsequent Tukey’s *post hoc *analysis revealed a reduction in locomotor activity of the 50 μM treated larvae when compared to their respective within group control at least *p* < 0.001. There was no statistical significance observed between 50 μM [WT/WT vs *sa17298/WT*] at *p* = 0.3117.

In Fig. 7d, larvae pre-treated in VPA for 2 h displayed an overall significant effect of genotype [*F* (1,60) = 33.15, *p* < 0.0001] and treatment [*F* (1,60) = 23.57, *p* < 0.0001] on larval locomotor activity with no significant interaction [*F* (1,60) = 0.1149, *p* = 0.7358] observed when a two-way non-RM ANOVA was performed. Tukey’s *post hoc *analysis showed statistically significant effect of VPA to decrease larval locomotor activity across groups at least *p* < 0.01 when compared with untreated larvae of the same genotype. However, VPA was unable to reduce the hyperlocomotion of *sa17298/WT* larvae to a comparable level as the VPA-treated WT/WT group (*p* = 0.0023).

### Effects of Neuroactive Drugs on the Behaviour of WT and Heterozygous *sa17298* Zebrafish Larvae in the Light-Dark Test

**Fig. 8:**
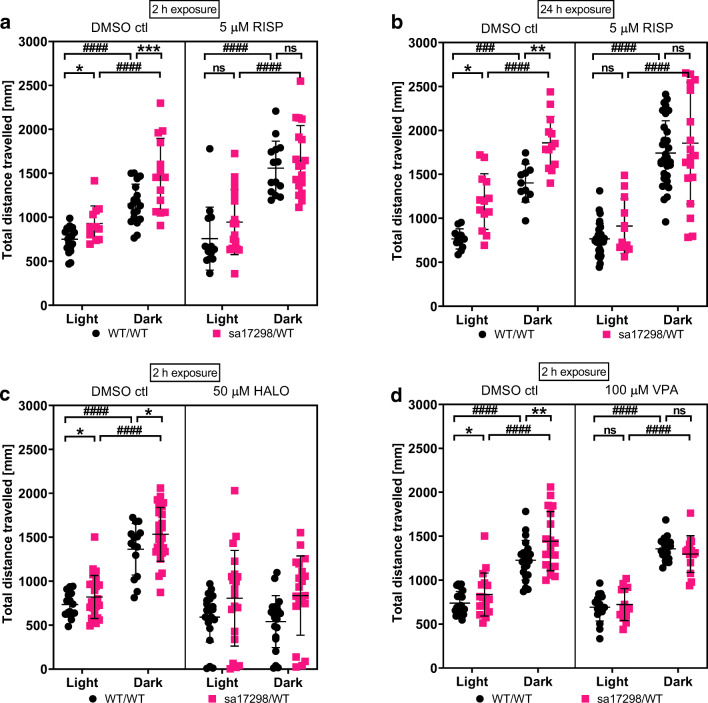
Effects of neuroactive drugs on the behaviour of 6-dpf WT and heterozygous *sa17298* larvae in the light-dark test. Larvae were exposed to different neuroactive drugs. Each dot represents individual larval measurement. Data analysed using two-way ANOVA followed by multiple comparison *t* test. Data represented as mean ± SD. **a** 2-h RISP, **b** 24-h RISP, **c** 2 h HALO and **d** 2-h VPA. ctl, control; HALO, haloperidol; RISP, risperidone; VPA, valproic acid. *p < 0.05, **p < 0.01, ***p < 0.001 [*cacna1da*^*WT/WT*^ vs *cacna1da*^*sa17298/WT*^] in respective groups. ^**###**^*p* < 0.001, ^####^*p* < 0.0001 [*cacna1da*^*WT/WT*^ vs *cacna1da*^*WT/WT*^] and [*cacna1da*^*sa17298/WT*^ vs *cacna1da*^*sa17298/WT*^]

The hyperlocomotor activity of heterozygous larvae relative to WT in the light and dark states was reversed after the exposure of larvae to all the treatment options i.e. 24-h exposure in 5 μM RISP and 2-h exposure in 5 μM RISP, 50 μM HALO and 100 μM VPA at *p* > 0.05. Larvae in either genotypes exposed to all the treatment groups but HALO were capable of eliciting a light-dark response by increasing their locomotion (*p* < 0.001) (See Fig. 8 and Suppl. Table 2).

### Effects of Neuroactive Drugs on the Behaviour of WT and Heterozygous *sa17298* Zebrafish Larvae in the Startle Response to Dark Flashes

**Fig. 9:**
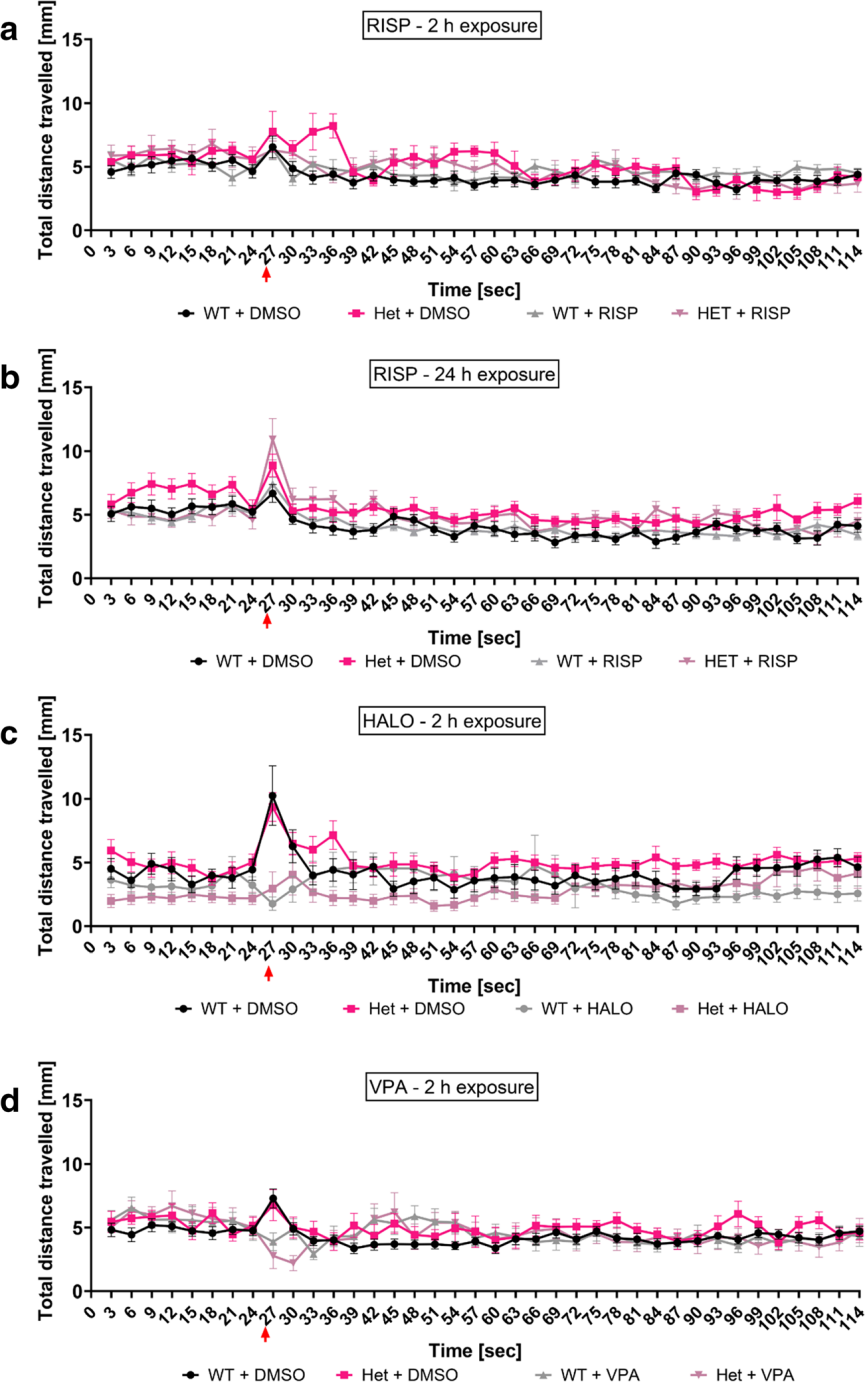
Time series graph of the effects of neuroactive drugs on the behaviour of 6-dpf WT and heterozygous *sa17298* larvae in the startle response to dark flashes test. Larvae were exposed to different neuroactive drugs. Each dot represents individual larval measurement. Data represented as mean ± SEM. **a** 2-h RISP, **b** 24-h RISP, **c** 2-h HALO, **d** 2-h VPA. HALO, haloperidol; RISP, risperidone; VPA, valproic acid

Larvae treated in vehicle control behaved similarly as their untreated (medium) counterparts previously described. In the 2-h exposure to RISP group, there was no difference in locomotor activity between WT and heterozygous mutant larvae prior to the onset of the dark flashes. After dark flashes onset, there was reduced startle response of heterozygous larvae in the first five dark stimuli with the next seven dark flashes resulting in comparable behaviour to the untreated group. In general, RISP at 2 h reduced heterozygous larval reactivity to the startle inducing dark flashes (*p* > 0.05, Fig. 9a). At 24-h exposure, RISP reversed the hyperlocomotor activity of heterozygous larvae prior to the onset of dark stimuli (*p* > 0.05, Fig. 9b). However, upon onset of dark flashes, heterozygous larvae behaved similar to their untreated siblings i.e. hyperactivity (*p* < 0.01, Fig. 9b). On the other hand, HALO was able to reverse the hyperlocomotor activity of heterozygous larvae both prior to and at the presentation of the startle inducing dark flashes (*p* > 0.05, Fig. 9c). Whereas, VPA treatment resulted in a delayed response of larvae to dark flashes and an overall reversal of hyperactivity of the heterozygous larvae (*p* > 0.05, Fig. 9d) (For modulatory activity of all drugs over time, see Suppl. Fig. 3 and Table 3).

### EEG Assessment

Student’s *t* test analysis of tectal EEG recordings of 6-dpf larvae indicated that there were no statistically significant differences with regard to the number of epileptiform-like discharges between *cacna1da*^*WT/WT*^ (*M* = 2.088, *N* = 12) and *cacna1da*^*sa17298/WT*^ (*M* = 4.333, *N* = 15); *t* (25) = 0.8741, *p* = 0.3904 (for example, see Suppl. Fig. 4).

## Discussion

In this study, we describe for the first time neurobehavioural changes in larval zebrafish with an essential splice variant mutation (*sa17298*) in the *cacna1da* gene. Since our mutant (*sa17298*) harbours a single point mutation (G > A) at the donor splice site of intron 20–21 of the *cacna1da-202* transcript variant, we anticipated that this could lead to dysregulation of *cacna1da* mRNA or protein expression, thus mimicking classical GOF or loss-of-function mutations. Agarose gel electrophoresis of rt-PCR products did not suggest exon skipping as a consequence of the mutation. The results showed significantly reduced “overall” cacna1da mRNA and 202 transcript levels in both heterozygous and homozygous mutants relative to WT. For the 201 transcript, while heterozygous mutants showed reduced levels, homozygous mutants on the contrary showed increased levels. We speculate that the *sa17298* splice variant mutation leads to a premature termination codon in variant 202, as a result of intron retention, thereby resulting in nonsense-mediated decay, thus resulting in loss or significant reduction of this particular transcript and subsequent compensation through an increase in levels of transcript variant 201.

Our study revealed significant behavioural impairments in *cacna1da*^*sa17298/WT*^ mutants when assayed for locomotor activity (under light conditions), during light-dark transition and in the startle response to dark flashes. However, we did not observe significant PPI deficits, thigmotaxis-related abnormalities or epileptiform-like discharges in larval brains as measured by EEG recordings.

The behavioural response of *cacna1da*^*WT/WT*^ in the light-dark stimulus task is in agreement with other studies [41, 56–59], which showed an abrupt increase in locomotor activity of larvae during rapid switching from light to dark. Kedra et al. [56] revealed that homozygous *tsc2*-deficient larvae (model of tuberous sclerosis complex, in which 90% of patients display seizures) exhibited the same behavioural response as described here for *cacna1da*^*sa17298/WT*^ mutants i.e. a pronounced increase in locomotor activity of *tsc2*^−/−^ larvae compared with their control counterparts in the dark phase of the light-dark stimulus task. *tsc2*^−/−^ larvae also displayed increased thigmotaxis as well as a preference to light phase in the light-dark preference task. Exacerbation of thigmotactic behaviour in zebrafish during dark phase is regarded as a marker of anxiety-like behaviour [42, 60], thus indicating an anxiety-like phenotype for *tsc2*^−/−^ larvae. In contrast, we did not observe changes in thigmotaxis between *cacna1da* genotypes. Thus, it is unlikely that the increased locomotor activity of *cacna1da*^*sa17298/WT*^ larvae in the dark phase of the light-dark stimulus task was a result of *cacna1da*-mediated anxiety-like behaviour.

Increased locomotor activity, as a readout of tonic-clonic-like seizures, was previously described in zebrafish genetic models of epilepsy [49, 61, 62], which correlated well with the occurrence of epileptiform-like discharges in larval brains as measured by EEG. In some human *CACNA1D* mutation carriers, different types of seizures have been observed (focal and/or generalised seizures) [17, 20, 63]. In our study, we observed epileptiform-like discharges in the EEG assay only in 3 out of 15 *cacna1da*^*sa17298/WT*^ larvae. Thus, it is unlikely that the hyperlocomotion displayed by *cacna1da*^*sa17298/WT*^ mutants is associated with seizures.

*CACNA1D* mutations have been implicated earlier in SCZ [8, 13, 21]. In behavioural pharmacology in rodents, the most common approach is to mimic psychotic-like positive symptoms by administering drugs that target N-methyl-D-aspartate (NMDA) (antagonism) or dopaminergic (agonism) receptors. These drugs induce psychosis in rodents with hyperactivity as a readout of this symptom (for review, see [64]). Interestingly, pharmacological studies revealed high correlation with regard to the ability of larval and adult zebrafish to mimic positive SCZ symptoms. For example, ketamine [65, 66] and MK-801 (also called dizocilpine) [54, 67] induce a robust increase in locomotion. Moreover, d-amphetamine or D_2_ receptor agonists elicit the same locomotor response in adult zebrafish [68, 69] as in rodents [70–74]. Previously, hyperlocomotion was observed in mouse genetic models of SCZ with mutations in *Disc1* [75] and *Nrg1* [76]. In larval zebrafish, this feature was also commonly observed in mutants harbouring mutations in different genes indicated by GWAS as SCZ susceptibility genes [77]. A study by Thyme et al. [77], which generated and analysed 132 different mutants of SCZ-risk genes, showed that hyperlocomotion was sometimes observed in a number of these fish.

To obtain further insight into the neurobehavioural changes in *cacna1da*^*sa17298/WT*^ larvae, we evaluated sensorimotor gating and habituation in larvae by performing two tests—i.e. ASR and PPI tests, as well as the startle response to dark flashes assay. The startle response to dark flashes assay, in which animals habituate to dark flashes when exposed to continuous light stimulus, allows for the assessment of non-associative memory [43, 48, 78, 79], which is also impaired in most schizophrenic patients [80, 81]. The ASR and PPI are the most commonly used tasks in SCZ research with an altered PPI response observed in schizophrenia patients [82–85], rodent [86–88] and some zebrafish models of SCZ [77]. The difference between PPI and startle response to dark flashes is the type of stimulus—i.e. auditory *versus *visual. As *CACNA1D* mutations have been previously implicated in deafness in humans [89, 90], mice [24] and even zebrafish [34], we assessed the hearing sensitivities of larvae across genotypes using different stimulus intensities (sound) and found them to be comparable. Nevertheless, no difference in PPI between WT and heterozygous mutants was observed, albeit with tendency of homozygotes towards enhanced PPI. The very low survival rate of homozygotes proved prohibitive with regard to our ability to collect and assay a sufficiently large set of larvae to reach statistical significance in our experimental setup. To the best of our knowledge, there is a lack of data as to whether *CACNA1D* mutation carriers are blind or have other visual deficits. However, a study reported *Cacna1d* knockout mice to have slight visual impairment but found no evidence that this affected their behaviour in the Morris water maze test (a “visual” spatial learning and memory task) [25]. In the zebrafish, *in situ *hybridisation analysis revealed expression of *cav1.3a* mRNA in the eye (although faint) and ear, while *cav1.3b* expression was evident in the otic vesicle of 5-dpf larvae. qPCR analysis indicated expression of *cav1.3a* mRNA both in the eyes and ears of adult zebrafish [27]. Taking into account that interlarval behavioural patterns were consistent throughout all experiments, we postulate that mutant visual acuity is preserved and that the behavioural phenotypes observed are a result of changes in brain activity and not visual disturbances. Although it appears that the *cacna1da*^*sa17298/WT*^ mutant response in this assay (startle response to dark flashes) might be related to cognitive symptoms of SCZ, this aspect of the mutant phenotype needs further investigation. Additionally, since the *CACNA1D* gene is duplicated in the zebrafish [27], the severity of the mutation may be attenuated through partial compensation by *cacna1db*. Thus, this may explain the absence of PPI deficits in the mutants.

We performed pharmacological profiling of WT and *cacna1da*^*sa17298/WT*^ larvae in the locomotor (under constant illumination), light-dark and startle response to dark flashes tests after exposure to different neuroactive drugs: two drugs used in the management of SCZ (HALO and RISP) and one drug (VPA) indicated for the treatment of epilepsy, and as add-on therapy in BP, migraine, anxiety or SCZ. The behavioural response of our WT fish after incubation with all 3 drugs is in agreement with previous studies which showed that all of them decreased locomotor activity in both larval and adult animals [35, 36, 54, 58, 91–93]. It seems that the response of WT larvae (i.e. hypoactivity) is a common feature for antipsychotics, as other drugs from this group also decreased baseline activity in WT larvae (e.g. droperidol, phenothiazine or clozapine) [94, 95]. With regard to VPA exposure, our study indicated that this drug substantially decreased activity of *cacna1da*^*sa17298/WT*^ larvae in all the three behavioural tests, although heterozygous mutants were significantly more active than their WT siblings in the locomotor test. The mechanism of action of VPA includes increasing levels of γ-aminobutyric acid (GABA) in the brain, inhibition of histone deacetylase and blockage of voltage-gated ion channels (sodium, potassium and calcium), such as CaV_1.3_ [96–98]. We therefore theorise that the effectiveness of VPA in decreasing locomotor activity in *cacna1da* mutants may act through a different mechanism other than seizure suppression.

In humans, *CACNA1D* mRNA has been detected in limbic and mesolimbic structures (i.e. amygdala, hippocampus, thalamus, hypothalamus, basal ganglia) [101, 102], which are enriched with dopaminergic neurons. Similarly, in mice, the *Cacna1d* gene is expressed in limbic and striatal areas [103–105]. Sidi et al. [27] observed that the expression of *cav1.3a* is detected as early as 30-h post-fertilisation (hpf) in the zebrafish brain (telencephalon, thalamic and hypothalamic diencephalon, midbrain, ventral hindbrain). From 48 hpf onwards, expression of *cav1.3a* becomes even more pronounced in almost the entire brain [27].

In conclusion, different zebrafish genetic models of epilepsy and to a lesser extent, SCZ, have emerged within the last 5 years (for comprehensive reviews see [106, 107]). Although the zebrafish brain is less complex than the mammalian one, fish are very sensitive to neuroactive compounds and their behavioural responses are easily tracked in 48-well plates. This allows for very rapid and efficient screening of compounds compared with rodents, thus enabling the identification of bioactive “hit” compounds for further investigation. Thus far, only zebrafish genetic models of epilepsy have been used successfully for discovering new therapeutic options in high-throughput screening assays [49, 108]. In SCZ research, there remains a need for identification of new antipsychotic agents with less side effects (especially those related to extrapyramidal symptoms) and effectiveness in alleviating negative and cognitive symptoms besides positive symptoms. Furthermore, with the advancements in genetic engineering methods in zebrafish [109–111], there is potential for using a precision medicine-based approach, where patient-specific mutations are introduced into zebrafish embryos to generate “customised” models for drug screening. This may aid scientists in tackling the pharmaco-resistance problem prevalent in SCZ patients, of which genetics plays a key role [see reviews [112, 113]. In relation to *cacna1da*^*sa17298/WT*^ larvae, our data appears promising, but further in-depth phenotyping is warranted to fully validate it as a new model of *Cacna1d*-mediated SCZ.

## Electronic Supplementary Material

ESM 1 (DOCX 5685 kb)

## Footnotes

1 It is important to note that gross morphological defects such as craniofacial abnormalities, smaller, fused or absent eyes, curved body axis, yolk sac necrosis and hyperpigmentation were visible as early as 2 dpf in a proportion of the offspring (~ 25%) when male and female adult heterozygous *sa17298* (obtained directly from ZIRC) were in-crossed. These morphological defects were present in heterozygous and homozygous *sa17298* populations as well as WT larvae (Suppl. Fig. 2). We therefore recommend that the line be outcrossed over multiple generations to remove other mutations that may confound the interpretation of results.
