# Supplementary material for: Zebrafish Larvae Carrying a Splice Variant Mutation in cacna1d: A New Model for Schizophrenia-Like Behaviours?
Source: Mol Neurobiol. 2020 Oct 14;58(2):877–94. doi: 10.1007/s12035-020-02160-5 (PMC7843589; doi:10.1007/s12035-020-02160-5)

| Table 1: List of primers for rt- and q-PCR | | | |
| --- | --- | --- | --- |
| Target name | Gene symbol | Forward primer | Reverse primer |
| *glyceraldehyde-3-phosphate dehydrogenase* | *gapdh* | 5′ GTGGAGTCTACTGGTGTCTTC 3′ | 5′ GTGCAGGAGGCATTGCTTACA 3′ |
| *ribosomal protein S18* | *rps18* | 5′ AGTTCTCCAGCCCTCTTATT 3′ | 5′ TCAACACGAACATTGATGGA 3′ |
| *cacna1da (amplifies both 201 and 202 transcript variants)* | *cacna1da* | 5′ TGCCACAAGCTGATAAACCA 3′ | 5′ CCTGCAGAAAGCACCCTTAT 3′ |
| *cacna1da (transcript variant 202)* | *cacna1da* | 5′ GGCTATGCAGATTATGTCTTCAC 3′ | 5′ TGGACGCAAAACTCTCAGAA 3′ |
| *cacna1da (transcript variant 201)* | *cacna1da* | 5′TACTTGGTTACTTTGACTATGCTTTCA3′ | 5′CTTTAGTCCTTTAGCTCTGTTTATGG3′ |

| Table 2: Effects of neuroactive drugs on larval behaviour in the light-dark assay: Two-way ANOVA test. HALO- haloperidol, RISP- risperidone, VPA- valproic acid. | | | | |
| --- | --- | --- | --- | --- |
| Treatment | Exposure time | ANOVA table | F (DFn, DFd) | P value |
| 5 µM RISP | 2 h | Interaction | F (1, 60) = 0.2889 | P=0.5929 |
|  |  | Illumination | F (1, 60) = 68.10 | P<0.0001 |
|  |  | Genotype | F (1, 60) = 2.294 | P=0.1352 |
| 5 µM RISP | 24 h | Interaction | F (1, 94) = 0.03859 | P=0.8447 |
|  |  | Illumination | F (1, 94) = 117.8 | P<0.0001 |
|  |  | Genotype | F (1, 94) = 2.125 | P=0.1483 |
| 50 µM HALO | 2 h | Interaction | F (1, 78) = 0.2112 | P=0.6471 |
|  |  | Illumination | F (1, 78) = 0.01388 | P=0.9065 |
|  |  | Genotype | F (1, 78) = 8.121 | P=0.0056 |
| 100 µM VPA | 2 h | Interaction | F (1, 58) = 1.046 | P=0.3107 |
|  |  | Illumination | F (1, 58) = 201.8 | P<0.0001 |
|  |  | Genotype | F (1, 58) = 0.1158 | P=0.7349 |

| Table 3: Effects of neuroactive drugs on larval behaviour in the startle response to dark flashes assay: Unpaired *t*-test. HALO- haloperidol, RISP- risperidone, VPA- valproic acid. | | | |
| --- | --- | --- | --- |
|  |  | P-value | |
| Unpaired *t*-test | Treatment | Before | After |
| WT vs sa17298/WT | 0.1 % DMSO 2 h | 0.0293 | 0.0444 |
| WT vs sa17298/WT | 5 µM RISP 2 h | 0.2059 | 0.7701 |
| WT vs sa17298/WT | 0.1 % DMSO 24 h | 0.0462 | 0.0047 |
| WT vs sa17298/WT | 5 µM RISP 24 h | 0.8743 | 0.002 |
| WT vs sa17298/WT | 0.5 % DMSO 2 h | 0.0345 | 0.4206 |
| WT vs sa17298/WT | 50 µM HALO 2 h | 0.1992 | 0.9147 |
| WT vs sa17298/WT | 0.5 % DMSO 2 h | 0.0357 | 0.0115 |
| WT vs sa17298/WT | 100 µM VPA 2 h | 0.3924 | 0.6849 |

Fig.1

Representative alignment of *cacna1da* transcript variants 201(v201) and 202 (v202) showing the primer sequences used for rt- and q-PCR.


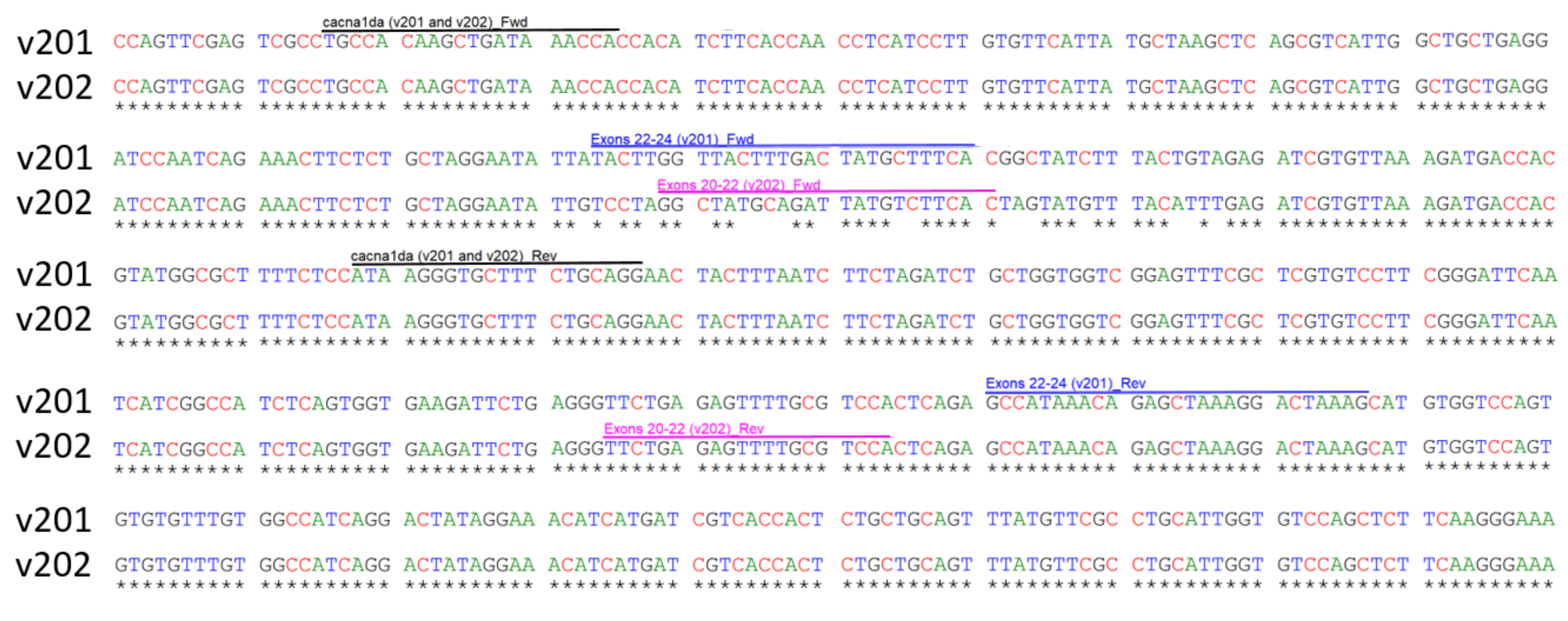


Fig. 2

Observable dismorphologies in 5 dpf larvae when adult (male and female) heterozygous *sa17298* (obtained directly from ZIRC) are in-crossed. Black arrow - craniofacial abnormalities, blue arrow – upward curved tail, yellow arrow – downward curved tail, purple arrow – pericardial edema, red arrow - small or fused eyes, hyperpigmentation and yolk sac necrosis. With some homozygotes looking indistinguishable from WT siblings. Scale bar (A-C): 1 mm.


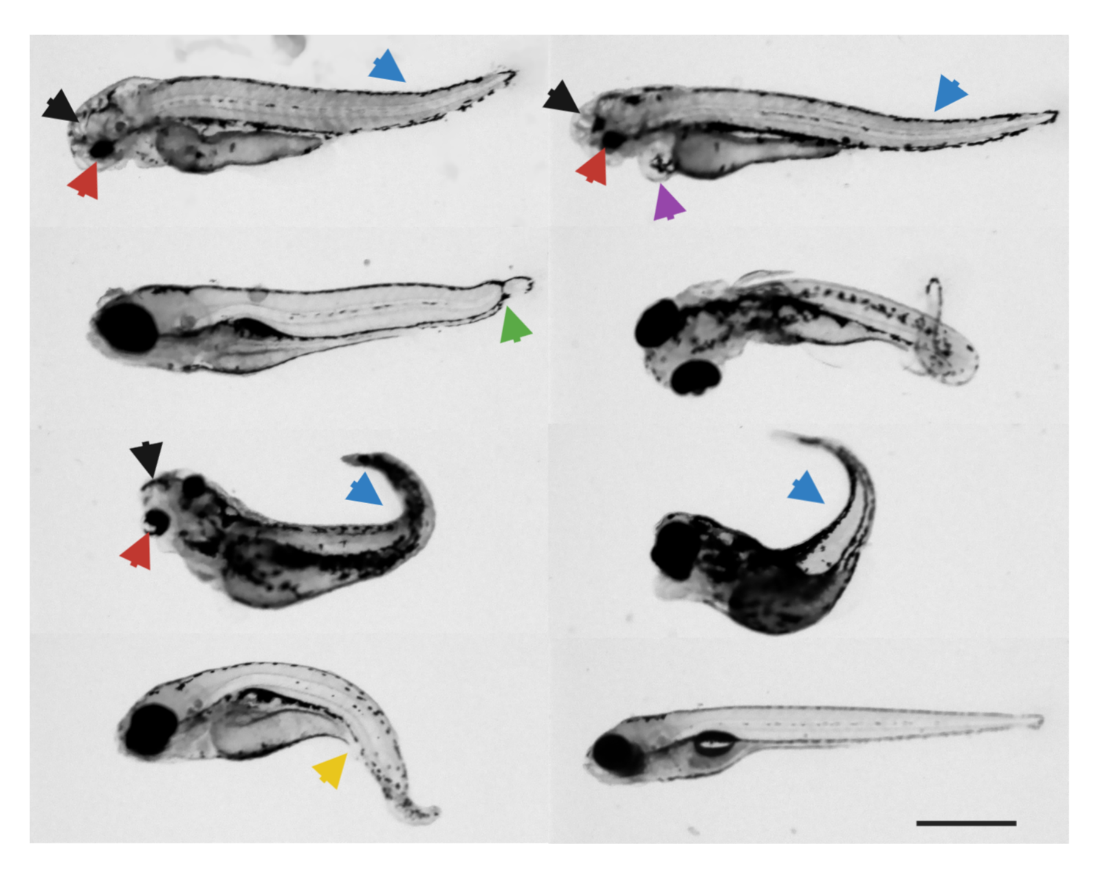


Fig. 3

Effects of neuroactive drugs on the behaviour of 6 dpf WT and heterozygous *sa17298* larvae in the startle response to dark flashes test. Larvae were exposed to different neuroactive drugs. Each dot represents individual larval measurement. Data analyzed using one-way ANOVA followed by multiple *t*-test. Data represented as mean ± SD. A) 2 h RISP, B) 24 h RISP, C) 2 h HALO, D) 2 h VPA. HALO- haloperidol, RISP- risperidone, VPA- valproic acid.

* p < 0.05 [*cacna1da^WT/WT^* vs *cacna1da^sa17298/WT^*] in respective groups

**^#^** p < 0.05 [*cacna1da^WT/WT^* vs *cacna1da^WT/WT^*] and [*cacna1da^sa17298/WT^* vs *cacna1da^sa17298/WT^*]


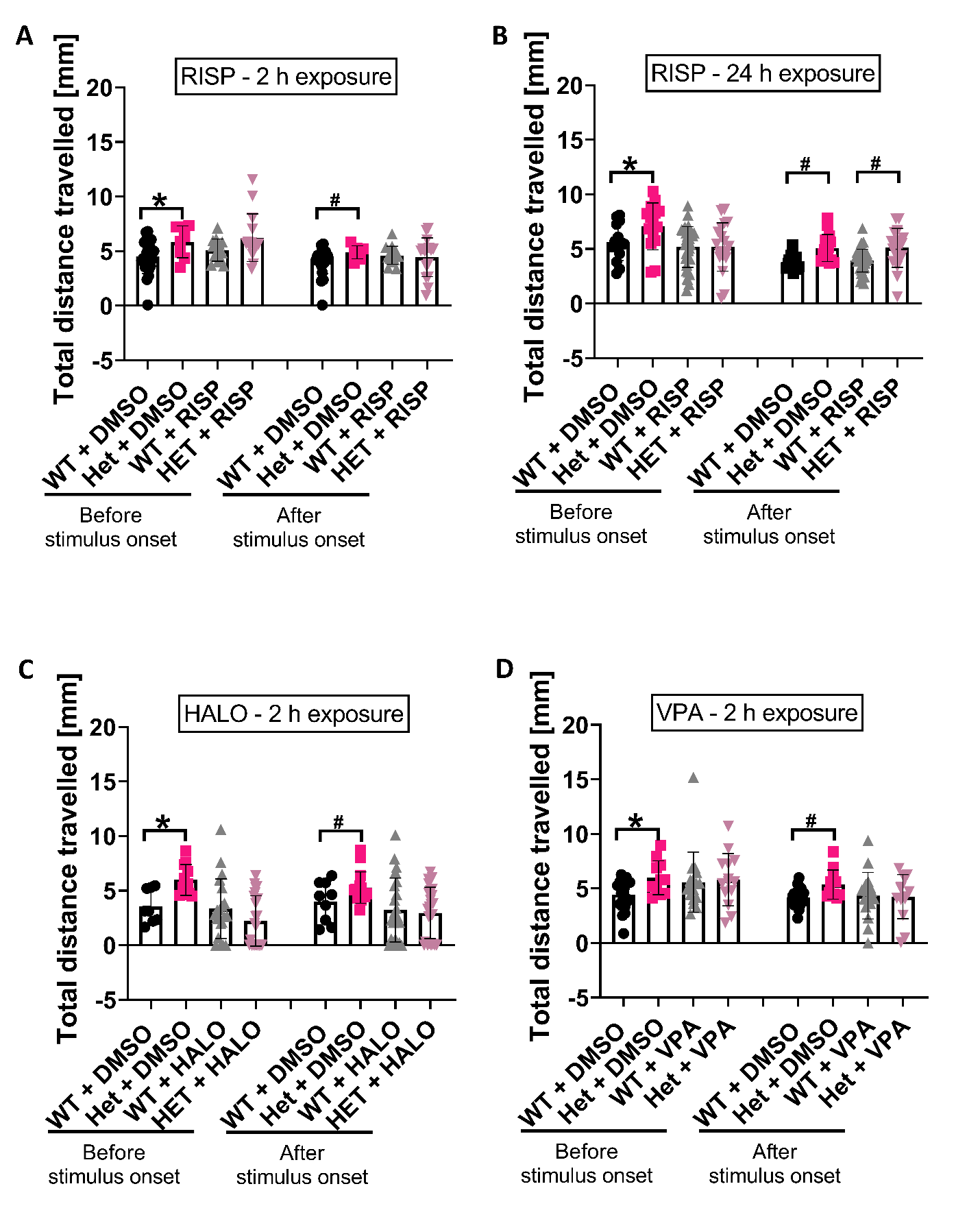


Fig. 4

Representative EEG recording showing the seizure-like discharges recorded from the optic tectum in zebrafish WT and sa17298/WT larvae at 6 dpf. A) Fragment of representative EEG recording from WT larvae. B) Fragment of recording from sa17298/WT larvae demonstrating the background and discharges.


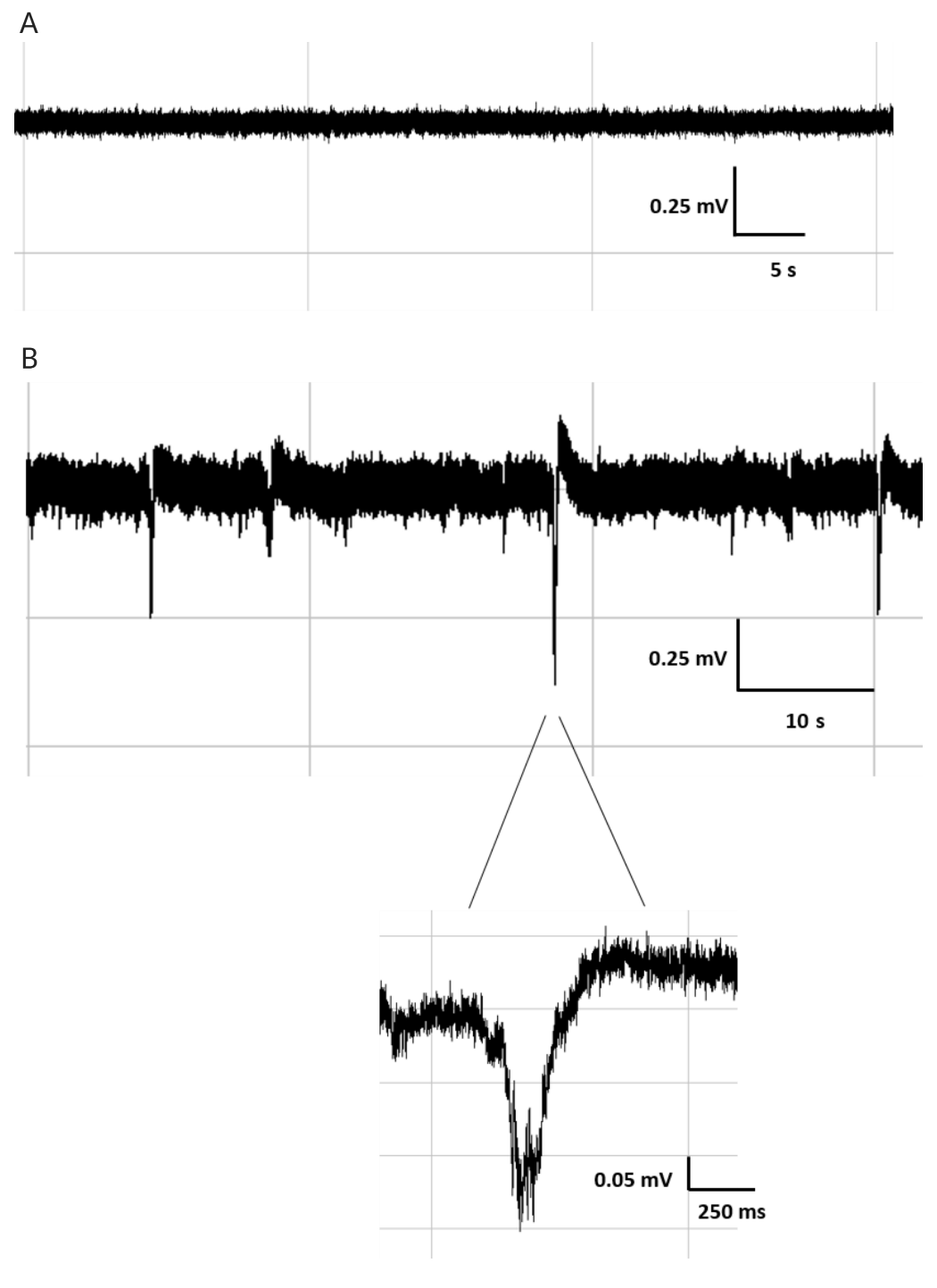

Supplement: Supplementary file 1 — (DOCX 5685 kb) [file 12035_2020_2160_MOESM1_ESM.docx]
